# Pregnancy and sexual related problems among women living on the street in dire Dawa City, Eastern Ethiopia, 2021: qualitative study

**DOI:** 10.1186/s12905-022-02006-3

**Published:** 2022-11-03

**Authors:** Daniel Tadesse Assegid, Legesse Abera, Meklit Girma, Mickiale Hailu, Bereket Tefera

**Affiliations:** 1 Midwifery department, Dire Dawa University, Dire Dawa, Ethiopia; 2 College of Health Sciences, Department of Midwifery, Mekelle University, Mekelle, Ethiopia

**Keywords:** Women, Street, Pregnancy, Sexual related

## Abstract

**Background:**

The problem of women and youths living on the street is a global phenomenon. It has created countless problems while they lived on the street; such as unwanted pregnancies, sexual exploitation and prostitution. It is a frequent observation to see women begging on the streets of major cities in Ethiopia having one or two babies by their sides. This study will give an in-depth understanding of the distressing and highly challenging problem among women on the street regarding pregnancy and related problems.

**Objective:**

To explore pregnancy and sexual-related problems among women living on the street in Dire Dawa city, Eastern Ethiopia 2021.

**Methods:**

A community-based phenomenological qualitative study was conducted at Dire Dawa city. Data was collected from homeless women and Key informants through focus group discussion and in-depth interviews using a semi-structured tool aided by a voice recorder. Data were analyzed thematically using computer-assisted qualitative data analysis software Atlas.ti7. The thematic analysis with inductive approach goes through 6 steps; Familiarization, Coding, Generating themes, Reviewing themes, Defining and naming themes and Writing up.

**Result:**

Women living on the street are more likely to experience physical abuse, sexual harassment, Sexual transmitted infection, unprotected sex and unwanted pregnancy. As a result, the fate of this pregnancy is abortion leads to high risk of getting health complications as most abortions are made in illegal and unsafe ways.

**Conclusion:**

Teen pregnancy, STI, rape and unsafe abortion are the major concerns as it accompanied by their homelessness situation and abusive lifestyles. The regional health bureau needs to understand the extent of problem and plan to organize an awareness creation program on STI, risk of pregnancy and SRH services.

## Background

The term “street” refers to places where individuals try to make a living, such as bus/car stops, outside of stores, near churches/mosques, roadways, parking lots, and other public spaces. Living on the street is a serious societal and individual problem that is fast spreading in developing nations as a result of social and familial issues such as poverty, domestic violence, physical and sexual abuse, and HIV/AIDS. The issue of homeless women and youths is a worldwide issue. While they were living on the street, it caused a slew of issues. Youth in shelters and on the streets are far more likely to become pregnant than those in families. They had the greatest lifetime rates of pregnancy, followed by youth in shelters and youth in their own homes [1–3].

Women who live on the streets are less likely to receive essential reproductive health care services, such as residing in the poorest part of the population, giving birth, and caring for their children away from the streets. As street people, girls and women, in particular, are subjected to sexual exploitation, rape, and prostitution, they are also at risk of unplanned pregnancies and reproductive disorders [4–6].

When homelessness is present, the situation is exacerbated since it increases sensitivity to the problem. The rate of rape-induced pregnancy among homeless women is substantially greater than the overall population, and it appears to be increasing as their housing situation becomes more unstable. Women begging on the streets of Ethiopia’s major towns with one or two babies at their sides are a common sight [2, 3].

The general social, psychological, economic, and health-related situations surrounding street women’s pregnancy practices and accompanying coping methods are poorly understood. Even the limited research available does not provide a complete picture of the situation on the ground.

As a result, this study was started in order to better understand pregnancy and sexual-related issues, in order to create useful data that could aid policymakers in developing suitable reproductive health programs for this disadvantaged segment of the population. This is the nagging issue which this study attempts to academically address.

**Research Questions**.

1. What are the pregnancy scenarios among women living on the street of Dire Dawa city?
2. What are the sexual-related problems among women living on the street of Dire Dawa city?

## Methods

### Study setting and design

A qualitative Phenomenology study design was conducted in Dire Dawa city from December 21 / 2020 – February 21 / 2021. Because the study topic was not studied at great length previously, we needed to have in-depth knowledge and understanding. As a phenomenology study emphasizes the lived experience of the participants related to the specific phenomena, this study gathered information from female street women regarding pregnancy and general sexual health-related problems.

Dire Dawa is one of the two chartered cities in Ethiopia that lies in the eastern part of the nation, on the Dechau river. There are 24 urban kebeles and 28 rural peasants associations under the chartered city.

According to the Dire Dawa Regional Health Bureau annual report, 2020/21 of MCH performance; the contraceptive acceptance rate is 32.6%, ANC 1 coverage 48%, ANC 4 coverage is 47%, syphilis and hepatitis test 89%, Skilled Birth attendant 67.8%, postnatal care coverage 61%, Teenage pregnancy is 25%, treatment of hepatitis and syphilis is 61% and 136% consecutively. Regarding the sexual and reproductive health education coverage for the last six months; health education was given to 627 individuals at the youth center, 44,759 individuals at the community level, 15,002 individuals at the school level, and 12,174 individuals at the workplace.

### Study population and sampling procedure

All reproductive age group women who are living on the streets of Dire Dawa city were the source population. In addition, key informants from relevant organizations were included as a source population. Overall, we recruited a total of 35; 14 IDI respondents, 3 key informants, and 3 FGD (6 members in each session) and they are included based on information saturation. A non-probability purposive sampling method was used to select women living on the street and key informants from the relevant organization such as PAD, LSAA and police station.

Street women who have resided on the streets of Dire Dawa city for a minimum of six months and above were included in the study, while mentally ill women who are living on the street were excluded from this study. Those mentally ill women were excluded with the help of a social worker (from PAD or LSAA) who usually works with them and knows one’s inability to respond correctly when we talk to them.

A survey was conducted with the help of officers from LSAA of Dire Dawa city administration and social workers from Positive action for development office to assess the areas/streets where most of the females are found. Since LSAA & PAD officers have experience in working with street women in various domains, Participants were selected by the researcher through the assistance of officers from LSAA of Dire Dawa city administration and social workers from PAD office.

### Data collection tool and procedures

Data was collected through focus group discussions and in-depth interviews. Both focus group discussions and in-depth interviews were conducted using a semi-structured interview tool aided by a voice recorder and key-note keeping. Participants (both women living on the street and key informants) were interviewed once and the interviews lasted about one hour. The interview was conducted at the shelter where the PAD office is provided and the compound of PAD office. Focus group discussions were carried out with 6 participants that lasted 80 − 100 min.

### Data processing and analysis

Data was analyzed thematically using computer-assisted qualitative data analysis software, Atlas.ti7. The thematic analysis with an inductive approach goes through 6 steps; Familiarization, Coding, Generating themes, reviewing themes, defining and naming themes and Writing up. The first step was familiarization; getting a thorough overview of all the data we collected before we started analyzing individual items. Data was transcribed by replaying the tape-recorded interview from in-depth interviews and focus group discussions. Their inductive meanings were extracted and described in narratives using the well-said verbatim of participants. Next, coding of the data was performed. Each code describes the idea or feeling expressed in that part of the text. Next, we look over the codes we’ve created, identify patterns among them, and start coming up with themes. The generated themes were reviewed to assure the accurateness and representations of the data. Defining and naming of themes were done, then finally the write-up of analyzed data was conducted.

### Result

Three FGD’s were conducted where each session consisted of 6 members, 14 homeless women and 3 key informants were included in the in-depth interview. As seen in Table 1, a total of 32 women living on the street participated in this study [Table 1].

As shown in Table 2; From the qualitative analysis of data, two major themes were driven. They are Pregnancy scenarios and sexual-related problems obtained from participants’ interviews and discussions. From each major theme, six subthemes were driven. Teen pregnancy, Nutritional problems during pregnancy, Sexual and physical abuse during pregnancy, Contraceptive issues, Misconception on risk of pregnancy, and Unsafe abortion/ self-induced abortion were derived from the pregnancy scenario. While Survival sex, STI, Child prostitution, Extra vaginal/ unprotected sexual intercourse, Physical and sexual abuse, Barriers to SRH service, were derived from sexual-related problems [Table 2].

**Table 1:** Socio-demographic characteristics of respondents

Characteristics	IDI	FGD
Age		
15–25	8	10
26–35	6	5
35–45	0	3
Education		
Non-formal educated	9	12
Primary and below	5	6
Secondary and above	0	0
Religion		
Muslim	9	13
Christian	5	5
Street life duration		
1–10 year	6	6
11–20 year	7	11
21–30	1	1

**Table 2:** Themes and subtheme of qualitative data

No.	Themes	Subthemes
1.	Pregnancy scenario	I. Teen pregnancy
		II. Nutritional problems during pregnancy
		III. Sexual and physical abuse during pregnancy
		IV. Contraceptive issues
		V. Misconception on risk of pregnancy
		VI. Unsafe abortion/ self-induced abortion
2.	Sexual related problems	I. Survival sex
		II. STI
		III. Child prostiituion
		IV. Extra vaginal/ unprotected sex
		V. Physical and sexual abuse
		VI. Barrires to SRH services

### 1. Pregnancy scenario

Eleven of the thirty-two women were pregnant at the time of the interview (between eight and 30 weeks gestation). The rest were those who have at least one previous pregnancy history in the past.

#### Teen pregnancy

This study found that the pregnancy rate among street women is very high, including under-age pregnancy. They become pregnant due to unwanted sexual intercourse. Most of them gave birth at an early age as mentioned below by one of the IDI participants.

“I gave birth to my first baby two years ago at the age of 15. A gang leader took me as his girlfriend and used to force me to have sex with him whenever he wanted. I used to get sick, vomit, and get confused during pregnancy. I never went to the hospital. My labor was intense and he took me to the hospital as I was screaming in pain…. I never wanted to live life like this. But at the age of 16, I am a mother of two.”

(women from IDI)

Pregnancy was the main problem for adolescents and youths who lived on the street. The discussant stated that women living on the street are exposed to unwanted sexual intercourse followed by an unwanted pregnancy. FGD participants discuss and share their experiences of how pregnancy is common among homeless teenagers.

“There is a risk of having a pregnancy on the streets. I was pregnant twice. The first was unwanted at the age of 14. I was so young. Two of my street friends got pregnant at the age of 14 and 15.”

(FGD discussant)

Another FGD discussant also shared her pregnancy story and mentioned her friend’s experience regarding pregnancy

“There are many of my friends who give birth on the streets and raise them on the road. I am now 17 years old, but I have 2 kids. So does my dear street friend. I did not understand anything about how a girl could now have a pregnancy while having sex with all our troubles and uncomfortable lifestyles.”

(A 17 years FGD discussant)

#### Nutritional problems during pregnancy

Participants from IDI stated that the unsuitability of their lifestyle with the responsibility of raising a child on the street was tough. In particular, pregnant teens emphasize facing countless hardships and danger on the streets. Also, they lacked access to proper food and clean water.

“While living on the street, I delivered one baby girl…..I am still raising my 8-month-old baby boy on the street and there are various problems when you raise a baby on the street” she replied in sorrow….” I am unable to produce sufficient breast milk to feed my baby as I don’t get proper foods to eat and pass it to my baby. Let alone wash and be clean, I don’t even have access to clean water for drinking. You know ………hmmmmm it is so hard to raise a kid here.”

(A women from IDI)

#### Sexual and physical abuse during pregnancy

On top of this, participants also mentioned that there is sexual abuse and exploitation during the pregnancy period. It is one of the problems affecting the physical, social, and psychological well-being of street teenagers during their pregnancy.

The story narrated by the IDI participant is presented as follows;

“Young homeless girls on the street often become pregnant. I am now eight months pregnant. When a street gang man found out I was pregnant with another man, he slapped me, punched me in my face, and left me to suffer. I am now living alone and discriminated against by his gang group. ….Again I was raped in my 6th-month pregnancy by 2 boys in the middle of the night. This life is so cruel. It is a scary place for any girl”

(women from IDI)

Another participant also mentioned her pregnancy experience as follows.

“………. 6 Months back again I gave birth to my second boy. ……. Since the gang leader knew it was not his baby, he stabbed me in my shoulder and another in my left leg. Life is not fair” she replied in sorrow and tears.

(women from IDI)

#### Contraceptive issues

One of the reasons stated for unwanted pregnancy found in the study was contraceptive issues. It includes unavailability of contraceptives, inappropriate use of contraceptives, and lack of information on the availability of the service providers.

“It is very risky. I mean the risk of pregnancy is much higher on the street because of unsafe sex and lack of awareness among the youth and adolescents. We don’t know where to find the protection for pregnancy. Besides, even condoms are expensive to buy. Children, such as 15 and 16 years of age are getting pregnant.”

(FGD discussant)

#### Misconception on risk of pregnancy

On top of the challenges that occur during pregnancy, there is an awareness gap regarding the risk of pregnancy. Most female adolescents living on the street have poor awareness of the risk of pregnancy. One of the IDI participants shared her awareness regarding the risk of pregnancy as follows.

“I think the risk of pregnancy on the street is not high. We don’t have enough food and a comfortable lifestyle on the street. I don’t think I can be pregnant at any time I have sex. The reason why I say this is, that I had sex multiple times on the street, mostly without my willingness; you can say on a daily basis, but I was only pregnant three times.”

(women from IDI)

Other participants from FGD also support this idea. She mentioned that the awareness among female adolescents of the risk of pregnancy with unsafe sexual intercourse was very poor.

“I think the risk of pregnancy on the street is high, but most female adolescents on the street don’t have awareness of the risk of pregnancy. They think that they can’t be pregnant at any time they have sexual intercourse. They think that if a woman is not in a comfortable lifestyle and she can’t get enough food, the risk of pregnancy is less likely. In general, especially teenagers and youths living on the streets are extremely exposed to the risk of being pregnant.”

(FGD discussant)

#### Unsafe abortion/ self-induced abortion

The finding indicates that unsafe abortion is highly practiced among women living on the street. As the respondent stated, female street women are more likely to experience early pregnancy due to rape and unprotected sexual intercourse while living and working on the street. As a result, the fate of this pregnancy is abortion, which makes adolescents at high risk of getting health complications as most abortions are illegal and unsafe. Participants have a misconception that pregnancy can be aborted safely by taking some kind of drug (ampicillin) with coca-cola. One of the participants from IDI shared her life experience as below.

“A month ago, I got pregnant again because some guy on the street raped me. I was so worried about how to overcome the pregnancy and raise another child on the street…….Then some girl told me about this medicine which would be taken with coca-cola to kill the baby inside my belly. She told me it was effective and it would kill the baby faster…..I took it. After a day, my stomach was in so much pain and heavy blood was coming out of my vagina. Then they took me to some traditional house and the traditional women gave me some drink which was squeezed from leaves….. I was in pain and bleeding for two days….”

(women from IDI)

The same story was narrated by another FGD participant as the following:

“….. 4 months ago, I got pregnant due to unwanted sexual intercourse. We went to some women who prescribe traditional leaves to abort the unwanted baby. I went there and she gave me some squeezed leaves. I drank that and after a day I went through so much pain around my stomach and blood started coming out of my vagina. I suffered a lot for 3 days. I smelled very bad. And the blood couldn’t stop. Finally, I fainted. When I woke up, I was in the hospital. I was told it was an infection as a result of the abortion. Finally, they gave me medicine and I spent 4 nights there. That’s how I survived.”

(FGD discussant)

All of the unwanted pregnancies end up with abortion and most of the abortions are unsafe in some sort of traditional way. One of the FGD participants mentioned her life experience.

“Almost every woman you see on the street was pregnant at least once. They usually abort it. Adolescents and children get pregnant. Most of the girls here either don’t have awareness of pregnancy risks or they are careless. I know one 16-year-old child who died after she got pregnant and inserted a traditional medicine inside her vagina. This is so sad. Even if we are living in the center of the urban at Dire Dawa, these things are happening.

(FGD discussant)”

### Sexual related problems

#### Survival sex

Street women and children don’t want to have a sexual relationship as they focus on fulfilling their basic needs like food and getting shelter. It was mentioned that teenagers are very exposed to unhealthy sexual relationships on the street for the sake of money and food. One of the participants from IDI narrates her opinion about sexual relationships on the street as follows.

“……….If your boyfriend is a dedicated street gang man, then you won’t die on the street, because they will treat you as part of their unit and part of their family. You just have to learn their little ticks, their little moments. We prefer the predictability of one man’s violence to the unpredictability of street violence. Protection and the never-ending need for money require sacrifices. The main thing is sexual favors.”

(IDI participant)

A similar result from the focused group discussion asserts the hardship level of living on the street without any protection. The only assured protection for them is to engage in some sexual activity even if they do not want it.

“Do you think I would open my leg to any man I get willingly? No, most of the sex here is forced and for money. For example, my first sexual act was rape. Most women on the street will have sex with street men to seek physical protection from any possible danger or attack while we live on the roads. They provide shields and protection. In return, we offer sex.”

(FGD discussant)

#### STI

This study found out that the other sexual-related problems for street children and women are STI and HIV/AIDS. Harsh and rough sex increases the risk of being infected with HIV. Female street children are affected by STIs as a result of sexual abuse. Also, not using a condom during sex puts them at higher risk of STI infection. The problem worsens as they don’t get appropriate treatment on time.

“I had STI problems more than 4 times on the street. There was a bad smell, an itching sensation, and fluid coming out of my vagina. A week ago, I was very sick with STI and I smelled very bad, even my friends were not willing to sit with me. I don’t even know where I caught the disease. And if you want to know everything, I have HIV in my blood. I confirmed that 5 years ago. (She cried badly). I don’t know where I got it. Most men do not want to use a condom when they have sex, even if I told them I had the virus.”

(women from IDI)

Besides the high case of STIs among women living on the street, it is also reported that there is a lack of awareness about STIs among female children who live on the street. One of the FGD discussants mentioned her experience as follows.

“It is a very high and ugly thing. I used to have signs like vaginal discharge and a bad smell coming out of my vagina….And most children on the street have that experience. But when we advise them to go to a health center, they refuse because they think that the smell is a result of uncleanness, as they don’t get enough water to get washed and keep themselves clean.”

(FGD discussant)

#### Child prostitution

It’s found that street girls practice commercial sex; they practice sex for money, food, and other material. Child prostitution is the main source of income for the member of the family/ guardian. As the discussant reveals, their elders and other street women force them to do sex for money.

"There is a lady named Ms. X who used to give me perfume and cosmetics to look good and make me work at night as a sex worker for drivers. I always inhale ‘Mastishe’ to forget and resist my pain. …….and she takes all of my money I brought after having sex with so many car drivers".

(16 yrs old discussant from FGD)

The above story is also supported by a narration from one of the key informants.

“The group leader, mostly a woman expects the young girls in the group to work for sex and bring back money to the group. The leading lady called Madam gives them small beauty products like perfume & lotions as a reward in exchange for sex money.”

(Key informant)

#### Extra vaginal/ unprotected sexual intercourse

It is reported that anal and oral sex are highly practiced among female children who live on the street.

“I go out for sex in exchange for money (for living and food), but people are very rude. Heavy truck drivers and drunken men ask us to do a very evil thing. They desired anus sex as well as oral sex. And most of them say that it is to prevent HIV and STDs. They think that the disease is only transmitted by vaginal sex.”

(Women from IDI)

Another participant from FGD narrate the same story.

“I was forced by one guy to have anal sex. When I refused, he slapped me and knocked my head hard. I became unconscious, and then all I remember was a burning pain in my anus. He left me on the roadside. When I woke up, I was bleeding heavily. My friends took me to Dil Chora hospital and I was hospitalized for 3 days.”

(FGD discussant)

Most participants from IDI share a common idea, which is that most of the street gang or the men they had sex with think that STI/HIV can only be transmitted through vaginal sex. Thus, they are forced to have unsafe sexual intercourse. The quoted narration from the participant of the study is presented as follows:

“Sometimes I slept with men for money. Even when I do business, I get raped sometimes. Some guys asked me to have sex in the anus; they asked us to suck their penis. At that time when I say no, they will force me to do whatever they wanted. Most of the sexual acts on the street do not involve the vagina. They do not want to fuck our vagina because they think the disease is transmitted only by vagina sex. So they forced us to do another evil thing.”

(17 year old IDI participant)

#### Physical and sexual abuse

Furthermore, this study revealed that gang rape, physical abuse and harsh sex are very common sexual-related problems among homeless women. Besides, participants who lost their virginity at an early age are exposed to psychological trauma. A woman from IDI narrated her experience as follows:

“If you want me to tell you about the day-to-day encounter ….trust me days won’t be enough. Let me tell you one story. I joined street life with one of my friends when I was 16 years old. One day, a random old guy called me and offered to invite me for food. As I was hungry, I accepted …..He then took me to a hotel and started showing me sex movies. He asked me the type of sex I would be willing to do and I replied “ I don’t want you to do anything. I just want to go out only.” He then had sex with me and took my virginity. He then called 2 other people to have sex with me afterward in the hotel. I was raped by a gang in Genaw hotel located in Gende Kore area. The 2 guys forced me to have rough sex……hmmmmmmm (crying) why do you remember me those devils?.... one of them raped me in my anus. I was ill for quite some time…”

(IDI pariticipant)

Another discussant from FGD also mentions her bad experience as follows;

“Yesterday, two guys came while I was sleeping with my children, then forced me to have sex. I refused so they beat me with a stone and one guy stabbed me on my hand. Here is the place where he stabbed ” showing her freshly dressed wound in pain. “I also went to the police station, but I dropped the case because they asked me if I had a witness and evidence. I was assaulted around 5 o’clock at night so there was no evidence.”

(FGD discussant)

#### Barriers to SRH service

The other striking finding from this study was about a sexual and reproductive health service issue. Sexual and reproductive health services have an important role for street females. However, participants mention that there are barriers that hinder street women from using the service. Among these, participants mentioned that service providers have an unwelcoming face in providing SRH services. Treatment of some healthcare professionals, fear of stigma and discrimination hinder them from visiting healthcare professionals. One of the FGD discussants shared her experience as follows;

“Let me tell you one story. It has been 3 months since I delivered a baby girl at the health center. At that time, the doctor yelled at me ‘why would you open your leg to have sex with everyone around with all your current problems?’ he shouted and treated me like a dog. Do you think I recklessly welcome everyone to have sex? No! Life is tough. So even if I wanted to go to a health center for some kind of case, I won’t go there because they humiliate me. Even last time I had an itching sensation in my vagina but didn’t go for a check-up. Yes, I know…… I have to go to a health center whenever I face a sexual-related problem, but I don’t feel good when I go there.“

(FGD discussant)

On top of this, this study reveals that street women have a lack of knowledge about the availability of the service. As the participants stated, they had never heard about SRH services. The lack of information on the availability of services is the biggest barrier to street children not using the service. Narration from a key informant mentioned as follows;

“As I told you, sexual-related problems like rape, harassment, unwanted pregnancy, and HIV are very high among women living on the street. So I believe there is a lack of knowledge on what to do when they are pregnant and also where to report any sexual abuse. There is still so much work to be done. They do not even know what a sexually transmitted disease is, especially children. But they are suffering from the diseases. There is a lack of awareness of where to go when they face sexual-related problems. Also, there is negligence among women living on the street due to different factors like substance abuse, rumors .......”

(key informant)

## Discussion

This study provided a chance to explore the past and present life experiences of women living on the street related to pregnancy and sexual-related problems. The finding reveals that women living on the street are highly exposed to teen pregnancy, sexual and physical abuse, unwanted sexual relationships, unprotected sex, and unsafe abortion.

Teen pregnancy is a common and a major concern as indicated by the unique pregnancy events that occur among women living on the street. Among the participants of this study, only 14 were currently pregnant during the interview and all of them had at least 2 pregnancy histories during their life on the street. Participants face countless hardships and dangers on the streets during pregnancy as it is accompanied by their homelessness, lack of food, clean water and adequate health care, although these are very essential for pregnant women. This finding is supported by other studies conducted among homeless women [7, 8].

The other striking finding of this study is sexual abuse during pregnancy among women living on the street. Street women are already the most vulnerable part of the community to sexual abuse on the street. These abuses continue while they are pregnant as well. The study participants mentioned that they are constantly under potential sexual predator attack whether they are pregnant or not. Our finding supports other qualitative research indicating sexual violence and physical abuse are part of their daily lives while in pregnancy. On top of the survival challenge of being pregnant on the street, they are impacted by physical, social discrimination, and mental well-being [1, 8].

In this study, lack of awareness of pregnancy risk is the other reason for the incidence of unwanted pregnancies among women living on the street. They have a misconception that lack of enough food and poor economic status won’t expose them to unwanted pregnancy. In contrast, this finding is not supported by other studies conducted in USA and East Africa; the participants from those studies are aware of pregnancy risk. This discrepancy may be due to the difference in the study population and the health care system in which they have a better awareness of the risk of pregnancy [1, 7].

As the pregnancy occurs among women living on the street is unwanted, the fate of this pregnancy is abortion, which makes adolescents at high risk of getting health complications as most abortions are made in illegal and unsafe ways. It is reported that most pregnancies go through unsafe abortions such as self-induced abortion and using harmful traditional ways. The result of this study is comparable to different studies worldwide conducted among homeless women in Kenya and USA, the studies reported youths as seeking or attempting self-induced abortions due to a lack of accessible abortion services [2, 5, 9, 10].

The other sexual-related challenges which our study participants experienced were similar to the findings of previous studies. In our study, homeless women experienced sexual assault, unsafe sex, and STI; such experiences were also identified in previous studies [12]. Our finding also confirms similar studies conducted in Ethiopian cities. In previous studies, homeless women were found to be vulnerable to rape, physical abuse, STI/HIV, and unsafe sex [3, 4, 6].

In this study, women living on the street did not get the most important sexual and reproductive health services for contraception, pregnancy, STI and safe abortion services. Homeless women suggested different reasons for not utilizing SRH services, such as; lack of awareness, some health professionals’ discouragement, fear of stigma and discrimination. The findings of this study are consistent with the previous qualitative studies carried out elsewhere. It was also reported that encountering stereotyping service and disrespect from healthcare professionals [11, 12].

The finding of this study indicates that unwanted sexual relationships or survival sex are the most mentioned coping mechanism among women living on the street. Youth experiencing homelessness reported that they or someone they knew was involved with survival sex–engaging in sexual intercourse in exchange for food, money, or protection. Such experiences were also identified in previous studies. Driven by poverty and the desire for protection from street gangs and unwanted attacks, many women and girls find themselves using sex as a commodity in exchange for protection, goods, services, money, accommodation, or other necessities. Such “transactional sex” involves sexual relationships, often with street gang leaders. It reflects men’s superior economic and physical protection position on the street and access to resources. Women’s difficulties in meeting basic needs in the most dangerous living environment require them to form symbiotic relationships that offer protection for them [7, 13, 14].

### Limitation of the study

Findings cannot be generalized to rural communities outside Dire Dawa city, while the study may have limited generalizability as it focused on one regional area (urban). Generalizations about the results are usually not able to be made because small samples are chosen and non-random sampling is used. Recall bias is the other limitation as some of the participants were unable to discuss clearly their past pregnancy scenarios.

## Conclusion

Women living on the street are vulnerable to physical and sexual abuse, STI, unsafe sexual intercourse, prostitution and teen pregnancy. As a result, the fate of this pregnancy is unsafe abortion, which makes adolescents at high risk of getting health complications. Lack of information on SRH service availability and encountering discrimination hinder them from visiting health institutions. In addition, lack of awareness of the risk of pregnancy and misconceptions about STI ways of transmission are more common in homeless women. The regional health bureau needs a plan to organize an awareness creation program on SRH services, including a health education program about STI transmission and the risk of pregnancy that should be provided.

## Data Availability

The data that support the findings of this study are available from Labor and social affairs agency of Dire Dawa Town but restrictions apply to the availability of these data, which were used under license for the current study, and so are not publicly available. Data are however available from the corresponding author upon reasonable request and with permission of Labor and social affairs agency.

## Declarations

For case of minors (those under the age of 18), or “emancipated minors”; I confirm that all the study protocols were approved by institutional review board of Dire Dawa University and ethical committee of College of Medcine and Health science. Informed consent form for those females who are under 18 years of age was obtained from the labor and social affairs agency of Dire Dawa city administration. Because under 18 adolescents need a parent/ guardian to make a decision with informed consent. Since they are homeless and have no parents, Labour and social affairs agency is the agency that is responsible and working with homeless children as a guardian. Hence, the ethical clearance for those under 18 children is given by labour and social affairs agency of Dire Dawa city administration after the ethics committee of Dire Dawa University approved this procedure and an official letter from Dire Dawa University is delivered to LSAA. The emancipated minors who are homeless without any guardian are allowed to give informed consent under the LSAA. According to FDRE (Federal Democratic Republic of Ethiopia) regulations, Informed Consent to participate is obtained from a legal guardian/parent, for “emancipated minors” approved by institutional review board of Dire Dawa University.

## Abbreviations

IDI: In-depth interview.
FGD: Focus group discussion.
PAD: Positive Action for Development.
SRH: Sexual and reproductive health.
LSAA: Labour and social affairs agency.
